# Cellular senescence and chromatin organisation

**DOI:** 10.1038/sj.bjc.6603636

**Published:** 2007-02-20

**Authors:** M Narita

**Affiliations:** 1 Cancer Research UK, Cambridge Research Institute, Robinson Way, Cambridge CB2 0RE, UK

## Abstract

Despite the potential importance of senescence in tumour suppression, its effector mechanism is poorly understood. Recent studies suggest that alterations in the chromatin environment might add an additional layer of stability to the phenotype. In this review, recent discoveries on the interplay between senescence and chromatin biology are overviewed.

Cellular senescence was first described as a state of permanent cell cycle arrest resulting from the replicative exhaustion of cultured normal diploid cells (Hayflick, 1965). Despite the static appearance and steady state of senescent cells, they are viable and metabolically active. Senescent cells exhibit a large and flat morphology with vacuoles, and an enlarged nucleus. Besides the morphological changes, the best-known marker is senescence-associated *β*-galactosidase activity (Dimri *et al*, 1995). More recently it has been shown that senescence is often accompanied by specific alterations of the chromatin structure, known as senescence-associated heterochromatic foci (Narita *et al*, 2003).

The senescence phenotype is extremely stable and, in contrast to quiescent cells (readily reversible cell cycle arrest), senescent cells are unresponsive to mitogenic stimuli such as serum or growth factors. Thus, senescence seems to be antithetical to ‘immortalisation’ in cultured cells and limits their neoplastic transformation. However, confirmation *in vivo* of this *in vitro* concept did not emerge until recently. A recent series of studies identified senescent cells *in vivo* using various models, thus reaffirming the significance of senescence as an intrinsic tumour suppressor pathway (Braig *et al*, 2005; Chen *et al*, 2005; Collado *et al*, 2005; Lazzerini Denchi *et al*, 2005; Michaloglou *et al*, 2005). Nevertheless, the molecular mechanism of senescence, particularly how senescent cells are driven into such a stable arrest, is not yet clear. To address this question, we have focused on the chromatin changes that occur during senescence and we have proposed that epigenetic regulation of gene expression might be involved in this process at least *in vitro* (Narita *et al*, 2003, 2006). Here the clinical significance of senescence as well as the role of chromatin alteration as an effector mechanism of senescence are discussed (Table 1).

## SENESCENCE AND AGEING

Originally, cellular senescence and organismal ageing were believed to be different concepts, yet it has been suggested that they are closely related owing to their shared ability to limit ‘lifespan’. Indeed, fibroblasts isolated from older individuals or patients with premature ageing syndrome such as Werner syndrome exhibit SA-*β*-gal activity earlier than those from young or healthy individuals, respectively. In addition, some senescence-associated genes, such as p53, can influence organismal lifespan. However, a direct causative effect of cellular senescence on ageing has never been shown. Now new studies shed light on this question; senescence may play a role in suppressing age-related cancer risk at the expense of juvenescence (Janzen *et al*, 2006; Krishnamurthy *et al*, 2006; Molofsky *et al*, 2006).

**Figure 1:**
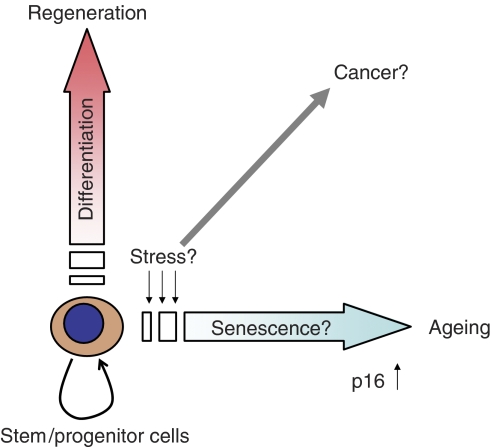
Balance between ageing and cancer. Age-dependent upregulation of p16 in stem/progenitor compartments might contribute to tumour suppression and ageing.

Ageing is associated with a reduction in the regenerative capacity of tissues, for which the functional progenitor cells are critical. An attractive idea is that senescence of the progenitor cells can be a cause of functional and physiological decline in tissue homeostasis and, as a consequence, individual ageing. A recent series of studies provided strong and direct insights into this senescence-ageing association (Janzen *et al*, 2006; Krishnamurthy *et al*, 2006; Molofsky *et al*, 2006). These reports showed that the age-dependent increase of p16 expression, an important marker as well as a mediator of cellular senescence, is associated with the limitation of self-renewal activity in the regenerative cells and contributes to ageing in bone marrow, brain and pancreatic islets (Janzen *et al*, 2006; Krishnamurthy *et al*, 2006; Molofsky *et al*, 2006). These studies raised the possibility that senescence of stem/progenitor cell compartments can, at least partially, be a direct cause of organismal ageing. The expression of the p16 tumour suppressor gene might balance the age-related risk for tumour development in stem/progenitor cell compartments (Figure 1).

## REPLICATIVE EXHAUSTION AND DNA DAMAGE-INDUCED SENESCENCE

The ‘replicative exhaustion’ that triggers senescence is essentially the erosion of telomeres. The telomeric regions found at the ends of chromosomes contribute to genomic stabilisation, and are shortened after each replication cycle. Once telomeres become critically short, they trigger senescence. Consistently, expression of telomerase, a reverse transcriptase that elongates telomeres, allows cells to proliferate beyond their normal replicative capacity and, accordingly, most cancer cells aberrantly express telomerase.

The senescence phenotype can be induced in early passage cells by a variety of cellular stresses, including DNA damage, oncogenic stress, oxidative stress and suboptimal culture conditions. Telomere-associated replicative senescence is often considered to be the ‘prototype’ of senescence and discriminated from other types of stress-induced senescence, which are telomere independent. However, the cause of telomere-associated senescence is attributed to the DNA damage response triggered by telomere dysfunction, and an intact DNA damage response is crucial for the induction of senescence (d'Adda di Fagagna *et al*, 2003; Herbig *et al*, 2004). Therefore, replicative senescence also boils down to stress-induced senescence.

Regardless of the trigger, senescent cells accumulate activity of the p53 and p16/Rb tumour suppressor pathways, both of which are commonly disabled in many cancers. It has been proposed that senescence can confer a physiological barrier to uncontrolled cell proliferation of damaged or aberrant cells, and therefore serves as intrinsic or therapeutic tumour suppressor machinery.

The clinical relevance of DNA damage-induced senescence was demonstrated by the studies that indicated that SA-*β*-gal-positive cells are present in tumours after chemotherapy, which causes DNA damage, in human breast cancer and in a mouse lymphoma model (Schmitt *et al*, 2002; te Poele *et al*, 2002). In the mouse model, DNA damage induces senescence in lymphoma tissues when the cells ‘cannot die’ owing to enforced expression of bcl-2, an antiapoptotic factor (Schmitt *et al*, 2002). Given the fact that immortalised cells, which ‘cannot senesce’, are very sensitive to DNA damage-induced apoptosis and that generally senescent cells are resistant to apoptosis, cells appear to have the ability to cleverly handle cellular insults based on a fine-tuned balance between apoptosis and senescence.

## OIS

**Figure 2:**
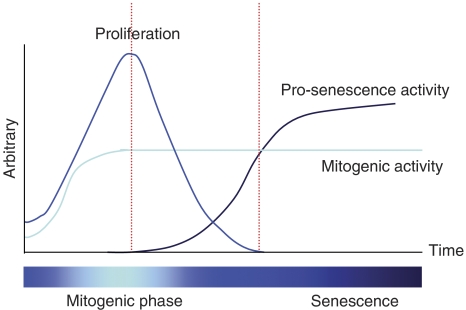
Oncogene-induced senescence (OIS). Constitutively active mitogenic stimuli induces rapid cell proliferation, but somehow the senescence machinery is triggered and eventually overcomes the mitogenic activity.

Among the stress-induced senescence phenotypes, oncogene-induced senescence (OIS) draws wider attention particularly after the recently emerging evidence of its clinical implications. Enforced expression of a constitutively active form of mutant *ras* promotes transformation in immortalised cells. However, the fact that oncogenic *ras* fails to transform normal cells and, paradoxically, can cause cell cycle arrest was known as an inexplicable phenomenon (Hirakawa and Ruley, 1988). Almost 10 years after this initial observation was made, more careful characterisation of oncogenic *ras*-induced cell cycle arrest lead to the identification of OIS, which is phenotypically indistinguishable from replicative senescence in normal diploid cells (Serrano *et al*, 1997). This process requires the intact mitogen-activated protein kinase (MAPK) pathway, the kinase downstream of ras. In marked contrast to other prosenescent stimuli, constitutively active mitogenic stimuli triggered by oncogenic *ras* initially cause rapid cell proliferation, which even accompanies loss of contact growth inhibition, a hallmark of cancer. This initial burst of proliferation is followed by the activation of at least two tumour suppressor pathways, the p53 and p16/Rb pathways, which counteract the mitogenic activity of *ras* and eventually override cell proliferation (Figure 2). In fact, the bypass of senescence in human cells requires inactivation of both the p53 and p16/Rb pathways (Serrano *et al*, 1997). The delayed kinetics of the accumulation of tumour suppressor gene products triggered by the abnormal mitogenic stimuli suggests that OIS is an intrinsic antitumour response that monitors cells to be sure that their proliferation is within an allowable range, although how cells determine the proliferation threshold and sense the deviation from it that triggers the execution of the senescent arrest is still unknown. Even if the theory makes perfect sense, the significance of senescence as a tumour suppressor mechanism *in vivo* remains controversial.

In 2005, a series of reports altogether provided strong evidence to support the clinical relevance of OIS as *bona fide* tumour suppressor machinery in various model systems in human and mouse. These studies identified senescent cells in premalignant or benign, but not in malignant, tissues such as *BRAF* (downstream effector of *ras*) associated naevi (better known as moles, benign tumours of melanocytes) (Michaloglou *et al*, 2005), *Kras*-associated lung adenomas or pancreatic intraductal neoplasias (Collado *et al*, 2005), AKT-PTEN-related prostatic intraepithelial neoplasia (Chen *et al*, 2005), and mitogenic E2F3-associated hyperplasia in pituitary glands (Lazzerini Denchi *et al*, 2005). Importantly, these oncogenic lesions result in full-blown cancers if they are combined with other mutations that disable the senescence machinery (such *p53*, *Rb* and *INK4a-ARF* locus mutations) in some model systems. These data suggest that an initial oncogenic lesion promotes hyperproliferation in the tissues, but subsequent provocation of OIS contributes to restrict tumour progression from a benign to a malignant state. Questions remaining to be answered include how generally benign tumours involve senescence machinery: do all benign tumours contain senescent cells and does OIS happen so often in our bodies as one can imagine from the example of moles (Michaloglou *et al*, 2005)? Ultimately, why are benign tumours benign?

In summary, cellular senescence, once suspected to be a cell culture artefact, is now more convincingly linked to pathophysiology of organismal ageing, DNA damage response and oncogenic stress.

## EPIGENETIC REGULATION DURING SENESCENCE

Generally, the senescence phenotype progressively accumulates over multiple cell cycles and senescent cells, much like differentiated cells, exhibit a specific gene expression profile. These observations suggest an active involvement of epigenetic gene regulation. Consistent with this view, certain types of cells exhibit senescent arrest that is accompanied by senescence-associated heterochromatic focis (SAHFs), a new type of facultative heterochromatin (Narita *et al*, 2003). Such drastic chromatin rearrangement can also be observed during some types of cellular differentiation, another state of stable cell cycle arrest (Francastel *et al*, 2000). Very little is known about the effector mechanism of cellular senescence, but the global chromatin reorganisation may not simply be a senescence marker, but rather play a key role in the senescence mechanism. In fact, there is a strong correlation between SAHF formation and the irreversibility of the senescence phenotype (Beausejour *et al*, 2003; Narita *et al*, 2003).

**Figure 3:**
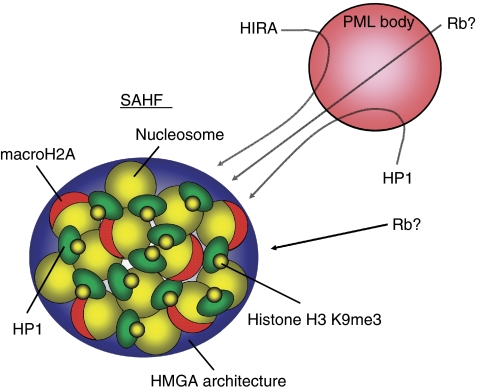
Model of SAHF formation. Senescence-associated heterochromatic foci contains a variety of chromatin proteins, such as K9-methylated histone H3 and HP1 (heterochromatic markers), macroH2A (histone variant) and HMGA proteins (architectural proteins). HP1 and HIRA (histone chaperon) transiently localise to PML bodies. Interestingly, Rb, which binds HP1, is also known to localise to PML bodies, although how PML bodies are involved in senescence is unknown.

The kinetics of the accumulation of SAHF-positive cells after triggering senescence by *ras* is well correlated with that of other indicators of senescence, such as SA-*β*-gal activity, p16 induction, Rb hypo-phosphorylation and cell cycle arrest; and SAHFs and DNA synthesis are mutually exclusive events (Narita *et al*, 2003). Senescence-associated heterochromatic focis are enriched for markers of heterochromatin, such as heterochromatin protein 1 (HP1) and Lys9 tri-methyl (K9me3) of histone H3 (which confers a docking site to HP1), and exclude euchromatic markers, such as histone H3 K9 acetyl and K4me3 (Narita *et al*, 2003) (Figure 3).

Interestingly, SAHF formation is largely dependent on the p16/Rb pathway in *ras*-induced senescence, although the impact of p53 on SAHF is marginal (Narita *et al*, 2003). p16 is an inhibitor of D-type cyclin-dependent kinases (CDKs). CDKs phosphorylate the Rb family of proteins, which are negative regulators of E2F transcription factors and, as a consequence, release the repression of E2F-target cell cycle genes. Knockdown of p16 or Rb significantly suppresses SAHF formation after *ras* introduction into human diploid fibroblasts, yet those p16 or Rb-deficient cells are still arrested and exhibit the prototypic senescence morphology and SA-*β*-gal activity. Interestingly, these SAHF-negative ‘senescent cells’ show deregulation of some cell cycle genes, indicating that there is an uncoupling of cell proliferation and expression of cell cycle genes (Narita *et al*, 2003). Thus, p16/Rb links SAHF formation and cell cycle gene silencing. Consistent with this, heterochromatin markers are accumulated on the promoters of the cell cycle genes during senescence (Narita *et al*, 2003; Rastogi *et al*, 2006). It is particularly worth noting that similar heterochromatinisation of some E2F-target genes was observed in a differentiation model (Ait-Si-Ali *et al*, 2004). These findings lead to a model in which active epigenetic rearrangement results in an alteration of the gene expression profile that contributes to the establishment and maintenance of new phenotypes during senescence and differentiation.

## CHROMATIN ASSEMBLY AND SAHFS

Adams and co-workers further characterised SAHFs and showed that macroH2A is enriched in SAHFs (Zhang *et al*, 2005) (Figure 3). The macroH2A is a transcriptionally repressive variant of histone H2A and, in female mammals, a marker of the inactive X (Xi) chromosome, which is a form of facultative heterochromatin (Costanzi and Pehrson, 1998). In fact, in *ras*-induced senescent cells, Xi is indistinguishable from other DAPI-staining foci, although a substantial difference in the components of SAHFs and Xi has been noted, where Xi is enriched for Polycome Group proteins and histone H3 K27me3 (which provides a docking site for Polycomb proteins), rather than HP1/histone H3 K9me3 (Plath *et al*, 2003). Further understanding of the commonalities and differences between SAHFs and Xi might confer insights into the structural and functional diversity of heterochromatin. It also remains to be elucidated how macroH2A contributes to the senescence phenotype and how, if at all, macroH2A is involved in the tumour suppressor machinery.

In addition to macroH2A, the authors also showed that histone chaperons, Asf1a and HIRA, play a critical role in SAHF formation, although the precise molecular mechanism is unknown (Zhang *et al*, 2005) (Figure 3). Interestingly, during senescence, HIRA and HP1 dynamically localise to promyelocytic leukaemia nuclear bodies (PML bodies), another senescence effector. Currently, the exact function of PML bodies and how PML bodies are involved in the senescence programme is not clear, but it has been suggested that PML bodies are sites for macromolecular assembly and posttranslational modifications of proteins. Accordingly, it has been speculated that the transient localisation of HP1 might be a prerequisite for the subsequent accumulation of HP1 in SAHFs (Zhang *et al*, 2005). The histone chaperones might also be involved, either directly or indirectly, in the nucleosome re-assembly during senescence, such as macroH2A deposition.

## CHROMATIN ARCHITECTURE AND SENESCENCE

More recently, another twist came from the biochemical analysis of chromatin-associated proteins in senescent cells (Narita *et al*, 2006). The experiments revealed that high-mobility group A (HMGA) proteins are senescence-associated chromatin binding proteins and that HMGA proteins are essential structural components of SAHFs (Figure 3). Furthermore, HMGA-dependent SAHF formation contributes to the stable senescence arrest. Interestingly, SAHF-positive cells also lose linker histone H1, which is known to compete with HMGA in binding DNA (Funayama *et al*, 2006).

The HMGA1 and HMGA2 proteins are non-histone architectural chromatin proteins, and have three ‘AT-hook’ domains that are responsible for binding to the minor groove of AT-rich DNA sequences (Reeves, 2001). HMGA proteins are not transcription factors *per se*, but have so-called ‘architectural transcription factor’ activity, which is associated with their ability to facilitate assembly of the ‘enhanceosome’ and typically create an ‘open’ chromatin environment conducive to transcription.

Moreover, *HMGA* has been linked to cellular proliferation and is known as an oncogene (Mantovani *et al*, 1998; Reeves, 2001). For example, expression of HMGA is induced by growth factor or serum stimulation (Lanahan *et al*, 1992). Both *HMGA* genes are highly expressed in proliferating cells in the embryo or in many tumours, and are downregulated upon cellular differentiation (Zhou *et al*, 1995; Reeves, 2001). Furthermore, HMGA proteins can promote tumorigenicity both *in vitro* (Takaha *et al*, 2004) and *in vivo* (Fedele *et al*, 2005, 2006b), and gene amplifications and translocations of *HMGA* genes occur in many human cancers (Reeves, 2001).

Therefore, the specific accumulation of HMGA proteins on chromatin during senescence and the functional association of HMGA with the senescence phenotype were initially puzzling (Narita *et al*, 2006). Although, *HMGA* can be induced by mitogenic stimuli, the upregulation of *HMGA* during *ras*-senescence is not a natural consequence of the constitutively active ras-MAPK cascade, as *HMGA* upregulation and the accumulation of the gene products on chromatin can be induced by other senescence-inducing stimuli, such as DNA damage and replicative stress. In addition, the kinetics of HMGA2 accumulation on chromatin during *ras*-senescence is progressive and coincides with the timing of SAHF formation.

Strikingly, knockdown of HMGA1 abolishes SAHF architecture completely, whereas HMGA2 knockdown has a lesser impact. Interestingly, chemicals that bind the minor groove of AT sequences of DNA displace HMGA proteins from chromatin and dissolve SAHFs in senescent cells. Surprisingly, HP1 accumulation on the chromatin fraction persists after dissolution of SAHFs by removal of HMGA, suggesting that the condensation of higher order chromatin and the global accumulation of biochemical markers of heterochromatin might be separable events during SAHF formation. Consistent with this observation, overexpression of HP1 alone fails to induce SAHF formation. These data raise an interesting question; how relevant is the higher-order architecture of SAHF for the senescence phenotype? Although SAHF formation requires p16, depletion of p16 in senescent cells (after establishment of SAHFs) has little impact on SAHFs as well as E2F-target gene expression, indicating that p16 is only required for establishment, but not for maintenance of SAHFs (Narita *et al*, 2006). However, p16 knockdown can de-silence some E2F-targets if SAHFs are dissolved by depletion of HMGA1, suggesting that HMGA1 may contribute to the silencing of these genes, at least in part, by regulating higher-order chromatin structure to modify the accessibility of transcriptional regulators. In accordance with these data, codepletion of p16 and HMGA1 causes a higher incidence of senescence bypass, demonstrating a cooperative effect of HMGA1 and p16 on stable senescence arrest.

These data indicate that HMGA proteins may be both pro- and antioncogenic, depending on the cellular context. Consistent with this view, both *Hmga1* transgenic mice and *Hmga1* knockout mice develop haematologic malignant tumours (Fedele *et al*, 2005, 2006a). Interestingly, *HMGA2* is located near the *HDM2* and *CDK4* loci on chromosome 12q13–15 and is often coamplified with both *HDM2* and *CDK4* (Berner *et al*, 1997), which target the p53 and p16/Rb pathways, respectively. Indeed, overexpression of *HDM2* and *CDK4* can cancel the HMGA2-induced senescence at least *in vitro*, although HMGA2 does not add further proliferative advantage to the *HDM2*- and *CDK4*-expressing cells. Furthermore, when the senescence programme is completely abrogated by E1A oncoprotein, ectopic HMGA2 promotes transformation activity of *ras* both *in vitro* and *in vivo* (Narita *et al*, 2006).

How exactly HMGA can be oncogenic and tumour suppressive depending on the cellular context is unknown. Interestingly a recent paper indicates that in tumour cells HMGA2 physically associates with Rb and activates E2F-target genes by inhibiting the function of Rb, a negative regulator of E2F-target genes (Fedele *et al*, 2006b). This is in marked contrast to the senescence setting where HMGA2 upregulation is associated with repression of E2F-target genes (Narita *et al*, 2006). Although it is yet to be tested if HMGA2 and Rb physically interact during senescence, understanding how HMGA2 has opposing activities on E2F-target expression depending on the context might give new insights into the role of chromatin architecture in senescence and tumorigenesis.

Deregulation of HMGA proteins in tumour tissues is not limited to malignant tissue, but in fact many benign tumours, particularly of mesenchymal origin, are accompanied by HMGA upregulation. Given the dual function of HMGA and the discovery of OIS in a benign context, it is very attractive to speculate that HMGA upregulation is an early event in response to oncogenic stimuli during tumorigenesis and contributes to the activation of the senescence programme. If this process is successful, tumours would remain benign and would not progress to a malignant stage. However, additional mutations that disable the senescence programme, such as disruption of the p53 and p16/Rb pathways, would promote tumour progression and reveal the oncogenic activity of HMGA.

### Chromatin alterations and *in vivo* senescence

Although *in vivo* validation of these individual components of SAHF is yet to be tackled, SAHF-like chromatin alterations were identified in some cases of *in vivo* OIS (Collado *et al*, 2005; Lazzerini Denchi *et al*, 2005). In addition, Schmitt and co-workers revealed a functional involvement of epigenetic regulation in OIS and tumour suppression *in vivo* (Braig *et al*, 2005). Using the E*μ*-N-*ras* transgenic mouse model, they showed that disruption of Suv39h1 histone methyltransferase, which is responsible for the histone H3 K9me3 heterochromatic mark, dramatically accelerates *ras*-induced T-cell lymphomagenesis. This data is consistent with the idea that the ras signalling could provoke the Suv39h1-dependent senescence machinery, which blocks *ras*-mediated tumorigenicity. Indeed, they also showed *in vitro* that *ras* expressing lymphoid cells exhibit a senescence phenotype with SAHF-like chromatin alteration, which is inhibited by *Suv39h1* knockout.

Furthermore, combined treatment of the same transgenic mice with inhibitors of DNA methyltransferases and histone deacetylases, enzymes that are associated with gene silencing and heterochromatin, showed a similar effect to that seen by disruption of Suv39h1 in *ras*-mediated T-cell lymphomagenesis. Although the precise mechanism by which epigenetic alterations are involved in antilymphomagenesis is unknown, and it is not clear to what extent we can generalise these observations, these data further our knowledge on the effector mechanism of senescence *in vivo*.

## CONCLUDING REMARKS

To date, treatment for cancer has focused on the removal and/or killing of cancer cells. Inducing senescence could be an additional therapeutic approach. Given the fact, however, that both the p53 and p16/Rb pathways, which are critical for senescence machinery, are often abrogated in tumours, targeting downstream events, such as chromatin alterations, would be more promising. Independent studies have identified different factors that are involved in SAHF formation (Figure 3). However, there is no insight into functional and structural association among those factors. Understanding how these individual factors contribute to senescence and tumour suppression as a network will be a challenge in the years to come.

## Figures and Tables

**Table 1:** Localisation and function of chromatin factors

	**Localisation and function**	
**Chromatin factors**	**General**	**Senescence**
HPI	Heterochromatin	SAHF component
H3 K9me3	Heterochromatin	SAHF component
MacroH2A	Heterochromatin	SAHF component
HMGA1/2	Chromatin architecture	SAHF component
Histone H1	Linker histone	Depleted
		
H3 K9 acetyl	Euchromatin	Excluded from SAHF
H3 K4methyl	Euchromatin	Excluded from SAHF
		
HIRA	Histone chaperone	SAHF regulation
Asf1a	Histone chaperone	SAHF regulation

HMGA=high-mobility group A; HP1=heterochromatin protein 1; SAHF=senescence-associated heterochromatic foci.
